# Surgical management of complicated arachnoid cysts: A case series

**DOI:** 10.1016/j.ijscr.2025.111856

**Published:** 2025-08-22

**Authors:** Hooman Koohestani, Behnaz Rahatijafarabad, Iman Attackpour Torik

**Affiliations:** a Department of Neurosurgery, Golestan University of Medical Science, Golestan Province, Iran; b Golestan University of Medical Sciences, Iran

**Keywords:** Arachnoid cysts, Subdural hematoma, Intracystic hemorrhage, Surgery, Complication

## Abstract

**Background:**

Arachnoid cysts are mostly benign, cerebrospinal fluid-filled sacs within the arachnoid membrane. Although they are often asymptomatic and discovered incidentally, they can also present with serious complications such as hemorrhage or rupture. This study aims to highlight the clinical presentation, radiological findings, and surgical management of complicated symptomatic arachnoid cysts in a case series.

**Case presentation:**

In this case series, we describe three patients with complicated arachnoid cysts: two children and one older adult. Although all three cases were initially managed conservatively, surgical intervention was eventually required due to worsening clinical symptoms. Surgical interventions included craniotomy with cyst fenestration in case one, subdural-peritoneal shunt in case two, and burr-hole drainage in the third. Presentations ranged from isolated headaches to focal neurological deficits. Outcomes varied: two patients had full clinical and radiological recovery, while one elderly patient passed away postoperatively due to myocardial infarction unrelated to neurosurgical complications.

**Discussion:**

These cases underscore the unpredictable nature of arachnoid cysts and how symptoms, age, and overall health can influence treatment plans and outcomes. Imaging plays a key role in diagnosis, while management must be carefully tailored to each case.

**Conclusion:**

These cases highlight the clinical variability and potential for complications from arachnoid cysts. Therefore, individualized management, taking into account patient age, characteristic symptoms, and comorbidities, is not just important, but essential for successful outcomes.

## Highlights

• Arachnoid cysts are often asymptomatic but may cause serious complications, including hemorrhage or trauma related issues.
• Management is guided by symptoms, cyst size, and location; conservative treatment is usually adequate for mild cases.
• The Galassi classification categorizes cysts by size and site, aiding in risk prediction and treatment planning
• When hemorrhage or mass effect occurs, surgical options such as craniotomy or endoscopic fenestration are required.

## 1 Background

Arachnoid cysts are sacs filled with cerebrospinal fluid that developed between the arachnoid membrane and the brain or spinal cord. They are commonly congenital; however, acquired forms may occur secondary to trauma, infection, or previous surgery. Arachnoid cysts are primarily benign and mostly found incidentally; however, some present significant neurological symptoms, for example, mass effect, rupture, or hemorrhage [1]. Post-traumatic hemorrhage or spontaneous hemorrhage into an arachnoid cyst is rare. However, it is a potentially life-threatening complication. Management of symptomatic arachnoid cysts, particularly hemorrhagic or ruptured, remains a subject of ongoing discussion in the neurosurgical community [2]. These reports present three unique and rare cases of symptomatic arachnoid cysts, highlighting the clinical relevance and uniqueness of the presented cases. These reports aim to discuss clinical presentation, imaging characteristics, and surgical vs. conservative management strategies based on existing literature. The work has been reported in line with the PROCESS Guidelines [3].

## 2 Case presentation

### 2.1 Case one

An 8-year-old boy with no significant medical history presented to the emergency department with progressively worsening headache and agitation. These symptoms developed over five days following a minor blunt head injury during a football match. There was no initial loss of consciousness at the time of injury, vomiting, or seizure activity. Neurological examination was regular. However, a physical examination revealed a noticeable prominence over the right temporal bone.

**Fig. 1:**
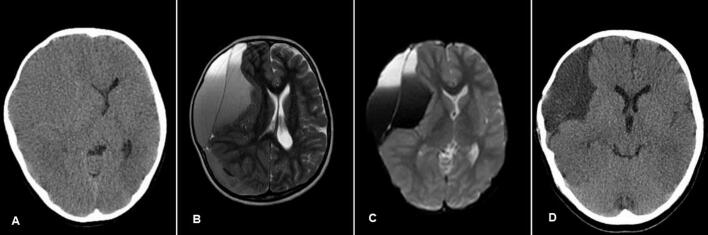
A) Preoperative non-contrast CT scan showing a large isodense lesion in the right frontotemporoparietal region, suggestive of an arachnoid cyst with possible hemorrhagic content. B) Preoperative MRI (T2-weighted) demonstrating a double-layered Sylvian arachnoid cyst with significant mass effect and compression of adjacent brain structures. The presence of fluid-fluid levels is indicative of subacute hemorrhage within the cyst. C) Another preoperative MRI revealed the tense outer cystic wall and internal hemorrhage with a mild displacement of midline structures. D) Postoperative non-contrast CT scan (6 months after surgery) showing significant reduction in cyst size with resolution of mass effect.

Computed tomography (CT) demonstrated a large iso-dense lesion in the right frontotemporoparietal region, accompanied by right temporal bone erosion and bulging, which has caused a mass effect. Magnetic resonance imaging (MRI) confirmed a double-layered Sylvian arachnoid cyst containing subacute hematomas (Fig. 1).

**Fig. 2:**
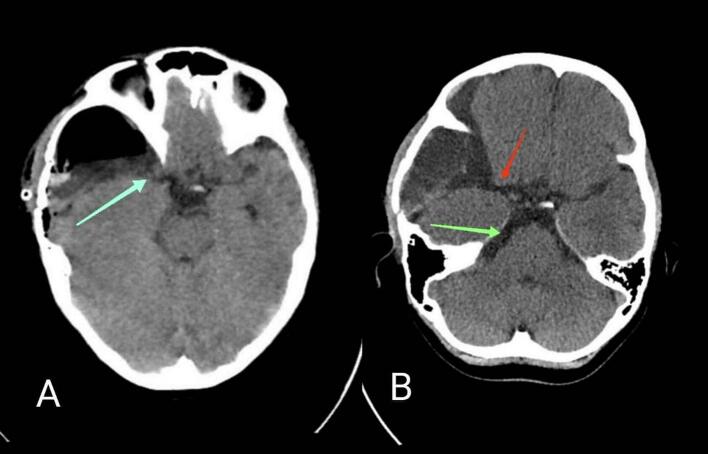
Postoperative and follow-up CT images of a third ventricular arachnoid cyst treated with endoscopic cystocisternostomy. A) Immediate postoperative CT scan showing the site of fenestration (blue arrow) between the arachnoid cyst and the basal cisterns. B) Follow-up CT obtained several months later demonstrates a patent communication (red arrow) between the cyst cavity and the basal cisterns, with clear visualization of cerebrospinal fluid flow in the basal cisterns (green arrow).

The patient underwent surgical intervention. A moderate-sized craniotomy was performed in the right frontotemporal region. When the dura was opened, the outer cyst wall was thick and tense. After incision, a yellowish liquid was released under high pressure. The outer cyst wall was subsequently excised and thoroughly irrigated. The inner cyst walls, in contrast, appeared non-tense and contained a low-pressure hematoma, which was carefully evacuated. Finally, communication between the cyst and the basal cisterns was established via cysto-cisternostomy (Fig. 2).

The patient's symptoms resolved completely after surgery. He was then placed on a postoperative care plan that included regular follow-up visits and imaging. Follow-up CT scans at one and six months confirmed full anatomical resolution, with no signs of residual lesions or recurrence. The patient remained symptom-free during the follow-up period, indicating a clinically and radiologically successful outcome.

### 2.2 Case two

A 77-year-old woman with a known history of hypertension and type 2 diabetes mellitus had been diagnosed with a right parietal arachnoid cyst during a previous evaluation for dizziness five years ago. She had been under regular checkups with CT imaging initially every six months then annually, showing a stable cyst size. She presented after a same-level fall at home and minor head trauma without loss of consciousness or acute neurological symptoms. Physical examinations were regular.

**Fig. 3:**
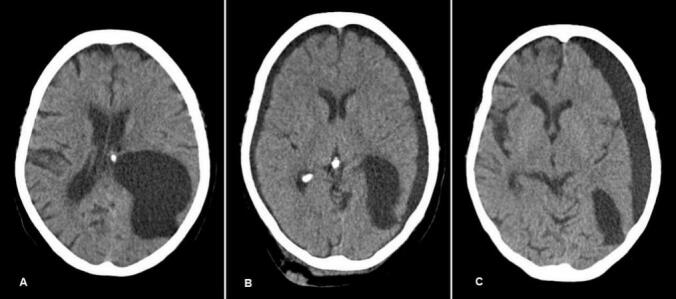
A) Pre-rupture: A large arachnoid cyst is visible, exerting a mass effect on adjacent structures. B) Post-rupture with bilateral subdural effusion: Immediately following the rupture, there is evidence of bilateral subdural effusion, likely due to cerebrospinal fluid leakage into the subdural space. C) Three months' post-conservative therapy: There is a persistent unilateral subdural effusion with a midline shift, suggesting incomplete resolution of the effusion but some degree of compensation.

Initial CT imaging showed no change in cyst morphology and a stable right parietal arachnoid cyst. Conservative management was planned for the patients. Six months later, the patient presented with a sudden-onset severe headache; although she remained fully conscious and neurologically intact, imaging revealed a rupture of the arachnoid cyst with bilateral subdural fluid collections, consistent with cerebrospinal fluid (CSF) leakage into the subdural space. Considering the patient's asymptomatic and stable neurological status, absence of raised intracranial pressure, and lack of acute symptoms, conservative management was chosen and continued (Fig. 3).

However, at her six-month follow-up, she reported progressive gait instability, generalized weakness, and right-sided hemiparesis. CT imaging was repeated, demonstrating a persistent unilateral subdural fluid collection that caused a mass effect. Given her worsening neurological status, surgery was planned, and a subdural-peritoneal shunt was placed. The patient underwent a standard preoperative cardiovascular evaluation, including an electrocardiogram (ECG) and cardiology consultation. No significant abnormalities were found.

While the surgery was technically successful and the patient showed improvement, she had a sudden cardiac arrest three days postoperatively. Despite efforts at resuscitation, she passed away. The presumed cause of death was myocardial infarction, potentially related to her underlying cardiovascular comorbidities.

### 2.3 Case three

A 13-year-old male without any previous medical history presented with minor head trauma accompanied by a headache. He reported an immediate onset of headache, described as diffuse, throbbing in nature, and rated moderate to severe in intensity. The headache was non-positional and was not associated with nausea, vomiting, visual changes, photophobia, or phonophobia. Neurological examination was routine, and there were no signs of meningeal irritation.

**Fig. 4:**
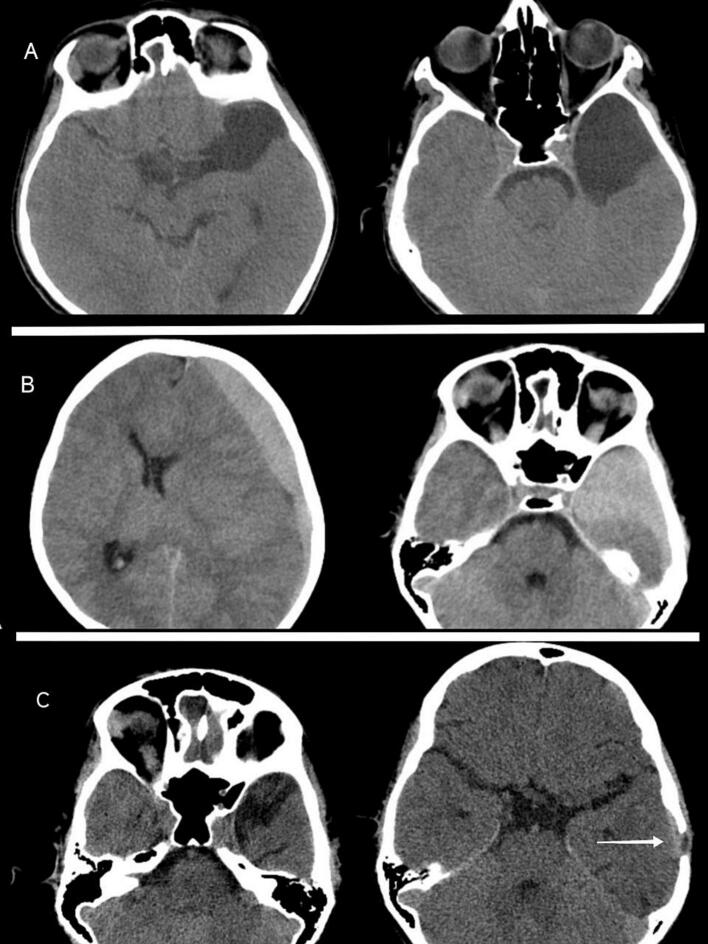
A) Initial CT: This axial non-contrast CT scan shows a hypodense, well-circumscribed lesion in the left middle cranial fossa, consistent with an arachnoid cyst (Galassi type II). No signs of acute hemorrhage, mass effect, or midline shift are present. The patient presented in a neurologically normal condition. B) Acute Deterioration, Hemorrhagic Complication: The previously hypodense arachnoid cyst is now partially hyperdense, indicating intracystic hemorrhage, a hyperdense crescent-shaped collection overlying the left cerebral hemisphere, consistent with a subacute subdural hematoma. A mass effect and midline shift are visible, including compression of adjacent brain structures. C) Postoperative CT: This three-month postoperative scan shows evacuation of both the intracystic hemorrhage and the subdural hematoma. The arachnoid cyst cavity appears decompressed. The brain parenchyma is relaxed, with re-expansion of the adjacent structures. No residual mass effect or new hemorrhage is noted, and the site of the burr hole.

Initial non-contrast head CT revealed a left middle fossa Grade II arachnoid cyst without evidence of intracranial hypertension (e.g., absent papilledema, normal optic disc margins). Conservative management was initiated due to the absence of elevated intracranial pressure signs. Three months after the initial evaluation, the patient was readmitted with a one-week history of worsening headache, nausea, and vomiting despite no recent trauma. Repeat CT demonstrated subacute hemorrhage within the cyst and a left frontotemporal subdural hematoma causing midline shift (8 mm deviation). No focal neurological deficits were observed during the physical examination (Fig. 4).

Emergency burr hole evacuation was performed to decompress the subacute subdural hematoma. Dark, liquefied blood was aspirated intraoperatively, and the cyst wall exhibited no active bleeding.

Postoperative recovery was uncomplicated, with symptoms resolved within 48 h. The patient was discharged neurologically intact. Three-month follow-up imaging and clinical assessment confirmed complete resolution of the hematoma, midline shift, and cyst-related mass effect, with no residual symptoms.

## 3 Discussion

Although arachnoid cysts can be found in a wide age range, they are most commonly diagnosed in the first decade of life. The male-to-female ratio for this phenomenon is 2 to 1, as males are affected more than females. Arachnoid cysts are found in about 1 % of adults, and over 94 % of them have no symptoms [4]. Arachnoid cysts are typically asymptomatic, but complications such as hemorrhage or rupture can happen after a head trauma or spontaneously [5,6].

Spontaneous hemorrhage in arachnoid cysts, though rare, has been reported in the literature. It is believed to occur due to rupture of fragile blood vessels around the cyst wall or within its base, even in the absence of trauma. As the cyst gradually enlarges by producing fluid, the increasing pressure may cause tearing of the wall, leading to bleeding into the subdural or intracystic space. Reported risk factors include minor head trauma, large cyst size, elevated intracystic pressure, and vascular fragility [7].

While these complications have been reported, the cases presented here are notable for their uncommon presentations, rapid deterioration, and distinct management challenges. Table 1 summarizes the clinical, radiological, and management characteristics of the three cases.

**Table 1:** Summary of the clinical, radiological, and management characteristics of the three cases.

Feature	Case 1	Case 2	Case 3
**Age/sex**	8-year-old boy	77-year-old woman	13-year-old boy
**Clinical presentation**	Headache, agitation after minor trauma	Dizziness history, later sudden headache, then gait instability & hemiparesis	Headache after minor trauma, later worsened with nausea/vomiting
**Imaging findings**	Right Sylvian cyst with subacute hematoma and mass effect	Stable parietal cyst initially; later bilateral subdural effusion; persistent unilateral collection with midline shift	Left middle fossa cyst (Galassi II), later developed hemorrhage and subdural hematoma with 8 mm midline shift
**Treatment**	Craniotomy, cyst wall excision, cysto-cisternostomy	Initially conservative; later subdural-peritoneal shunt	Emergency burr-hole evacuation
**Outcome**	Full recovery, symptom-free at 6-month follow-up	Death from cardiac arrest post-op despite neurological improvement	Complete resolution; symptom-free at 3-month follow-up

The differential diagnosis of arachnoid cysts includes dermoid cysts, hydatic cysts, epidermoid cysts, and brain tumors, which would be evaluated and diagnosed by further imaging modalities, including CT and MRI [8].

Considering the size and vast location the cysts can have, the symptoms vary from nausea, vomiting, headache, seizure, neurological deficit such as hemiparesis and cranial nerve impingement signs. In our cases, although case two was initially diagnosed by her dizziness, almost all of the cases were presented with signs after complication secondarily to mass effects including headache or gait instability, agitation, and hemiparesis. In Rashid et al. case report, a 7-year-old patient, one month after a head trauma, presented with photophobia, bilateral painful arm paresthesias, and intense agitation, which MRI showed bilateral arachnoid cysts with hemorrhage and evidence of transtentorial herniation [9]. Adin et al., in their case report, present two cases of intracystic hemorrhage associated with a subdural hematoma and mention that intracystic hemorrhage and associated subdural hematoma are common hemorrhage types that can happen spontaneously after exertion, physical activity, and trauma [10]. In our case report, we presented both isolated intracystic hemorrhage (case one) and subdural hematoma associated with intracystic hemorrhage (Cases two and three).

Arachnoid cysts are often located intracranial, but in some cases, they can be found around the spinal cord. Some studies have demonstrated that arachnoid cyst locations are the most important characteristic associated with symptoms and complications. Lesions in the middle cranial fossa and the retrocerebellar are the most common locations. Also, children and young adults and cysts larger than 5 cm have more significant risks of rupture and hemorrhage [5,6]. Of three complicated arachnoid cysts presented in our hospital, two were children. The Galassi classification categorizes arachnoid cysts into three categories based on location and size. Type I cysts (small, usually asymptomatic, located in the anterior, middle cranial fossa), Type II cysts (located superiorly along the Sylvian fissure, displace the temporal lobe), Type III cysts (large, take up the entire middle cranial fossa, displaces the temporal, parietal, and frontal lobes) [11]. In our case series, we observed that Type II cysts, which are located superiorly along the Sylvian fissure and displace the temporal lobe, can lead to significant mass effects, as seen in Case 3.

In our cases, there is no standard management protocol for ruptured arachnoid cysts. There is no explicit agreement on how to treat a ruptured arachnoid cyst. Treatment choice usually depends on where the cyst is and what symptoms the patient is experiencing. In cases of no or mild symptoms, conservative management is the choice, as the hemorrhage or hematoma can be resolved spontaneously. In the presence of seizure, mass effect, impaired CSF flow, or a decline in cognitive state, the surgery should be considered. The surgery options usually include burr-hole subdural drainage, craniotomy and cyst fenestration, and endoscopic cyst fenestration [5,6]. In our cases, an individualized treatment was implemented for the patient. All of the cases were initially treated by conservative management and observation, leading to surgery due to worsening symptoms. In the literature review that Adin et al. have done, only in 1 out of 30 cases was the thoroughly conservative approach reported successful [10].

There are two main surgical options for treating arachnoid cysts: open craniotomy and endoscopic fenestration. The choice depends mainly on the cyst's location and the surgeon's experience. Both methods seem to work equally well, though endoscopy usually has fewer complications and a quicker recovery. Craniotomy may be better for surface cysts, while endoscopy is often preferred, particularly when operating near vital or anatomically complex regions [12]. Although an open craniotomy was done for our first case due to the size and location of the lesion, in cases two and three, the decompression of the mass was done with a bur hole and subdural-peritoneal shunt. Cystoperitoneal (CP) shunting is another treatment for symptomatic arachnoid cysts that works by draining fluid from the cyst into the abdomen, helping to reduce pressure and symptoms. It has a low complication rate and is especially safe for older patients, though it can be used for people of all ages. Compared to other surgeries, it provides similar results but with fewer risks. The main downside is the potential for issues with the shunt, like infection or dependence. Despite this, it is considered a reliable option for treating these cysts [13]. Joen et al. report a two-month-old girl with an enlarging arachnoid cyst and worsening hydrocephalus, cystoperitoneal shunting was performed after failed craniotomy and fenestration. Follow-up MRI five years later showed a reduction in cyst size and resolution of hydrocephalus, highlighting cystoperitoneal shunting as an effective treatment for such cases [14].

A key limitation of this study is the small sample size. The low number of detected cases may be partly due to missed diagnoses in asymptomatic patients. Moreover, the sudden cardiac arrest in the second case prevented proper follow-up and restricted the evaluation of postoperative or long-term progress.

## 4 Conclusion

These cases illustrate the variable and unpredictable nature of arachnoid cyst complications. They emphasize the need for individualized management based on age, symptom severity, and risk profile. In conclusion, arachnoid cysts are typically benign intracranial lesions that rarely rupture. For asymptomatic cases, careful observation is generally in most cases sufficient. However, when complications such as hemorrhage occur, surgical intervention may be necessary.

## Consent

Informed consent was obtained from the patients for publication.

## Ethical approval

This study was approved by the relevant Ethics Committee.

## Guarantor

Behnaz Rahatijafarabad.

## Research registration number

This case series doesn't include any ‘First in Man’ procedure.

## Funding

The authors of this study declare that there are no funding sources.

## CRediT authorship contribution statement

**Hooman Koohestani:** Conceptualization, Methodology, Software, Supervision. **Behnaz Rahatijafarabad:** Data curation, Writing – original draft, Software, Validation, Writing – review & editing. **Iman Attackpour Torik:** Visualization, Investigation.

## Declaration of competing interest

There are no conflicts of interest to declare.
